# Electroactive Smart Materials: Novel Tools for Tailoring Bacteria Behavior and Fight Antimicrobial Resistance

**DOI:** 10.3389/fbioe.2019.00277

**Published:** 2019-10-18

**Authors:** Margarida M. Fernandes, Estela O. Carvalho, Senentxu Lanceros-Mendez

**Affiliations:** ^1^Centre of Biological Engineering, University of Minho, Braga, Portugal; ^2^Centre of Physics, University of Minho, Braga, Portugal; ^3^BCMaterials, Basque Center for Materials, Applications and Nanostructures, UPV/EHU Science Park, Leioa, Spain; ^4^Ikerbasque, Basque Foundation for Science, Bilbao, Spain

**Keywords:** electroactive materials, bacteria, antimicrobial resistance, physical stimuli, biomimetics

## Abstract

Despite being very simple organisms, bacteria possess an outstanding ability to adapt to different environments. Their long evolutionary history, being exposed to vastly different physicochemical surroundings, allowed them to detect and respond to a wide range of signals including biochemical, mechanical, electrical, and magnetic ones. Taking into consideration their adapting mechanisms, it is expected that novel materials able to provide bacteria with specific stimuli in a biomimetic context may tailor their behavior and make them suitable for specific applications in terms of anti-microbial and pro-microbial approaches. This review maintains that electroactive smart materials will be a future approach to be explored in microbiology to obtain novel strategies for fighting the emergence of live threatening antibiotic resistance.

## Introduction

Living cells respond to a range of chemical, biological, and/or physical signals, which individually or combined, are able to enhance cell target functions such as adhesion, proliferation, migration, and differentiation (Pérez et al., 2013). Biochemical cues are the most widely explored and proven signals to trigger specific biological responses (Sundelacruz and Kaplan, 2009). Among physical stimuli, mechanical forces are the most studied ones, being recognized to affect biological entities in a way that goes beyond the structural role that it plays to hold cells together (Paluch et al., 2015). In fact, the knowledge that cells and tissues are able to sense physical stimuli and translate them into biochemical and biological responses in a process called mechanotransduction, has been paving the way for the development of active materials with specific surface characteristics and surface patterns to be applied in regenerative medicine (Cartmell and Dobson, 2011; Bidan et al., 2018). Whereas, the mechanical forces are widely explored for triggering cells biological response, electrical, and magnetic cues are emerging as novel strategies (Pu et al., 2007; Qazi et al., 2014). The possibility of using electrical cues is based on the well-known cells' electrical properties. As an example, our sense of touch is transmitted to the brain via electrical pulses (Graczyk et al., 2016). Across the plasma membrane, an electrical voltage is also present, as inside the cell membrane the environment remains more negatively charged than the outside (Ghasemi-Mobarakeh et al., 2011).

While it has been demonstrated that mechanical, magnetic and electrical cues from the surrounding of mammalian cells influence their biological response, the potential of using these stimuli on bacterial cells has been largely overlooked. The biochemical environment surrounding bacteria has been the main cue reported to affect them. Indeed, bacterial chemotaxis is one of the best studied biological sensory systems in bacteria. A microfluidic gradient generator was proposed to study this phenomenon (Mao et al., 2003). The developed microfluidic assay stablished a gradient of chemoeffectors within the microchannel via diffusion between parallel streams of liquid in laminar flow, and the movement of cells was more effective toward the stream containing a chemoeffector rather than the one containing buffer. It was thus demonstrated that *Escherichia coli* (*E. coli*) possess chemotactic sensitivity and that cells respond to amino acid concentrations as small as a few nanomolar, when microfluidic devices are used.

Besides the biochemical stimuli, proven by bacterial chemotaxis, there have been evidences that bacteria can also feel their external environment in a similar way to that of mammalian cells (Persat et al., 2015), i.e., by responding to other physical signals.

**Figure 1:**
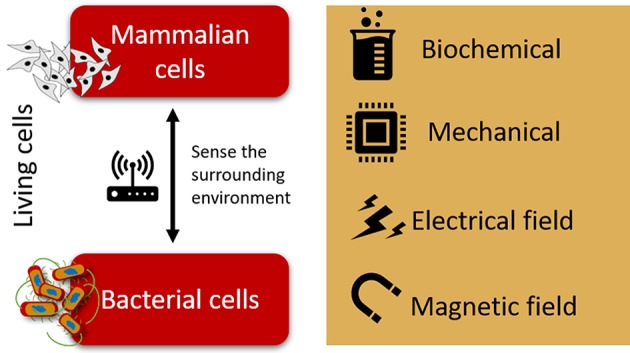
Schematic representation of the biochemical, mechanical, electrical, and magnetic cues to which mammalian and bacterial cells are sensitive.

This review is based on the possibility of using novel active materials for tailoring bacteria response by providing mechanical, electrical or magnetic cues in a biomimetic approach (Figure 1). Such strategies may trigger physical mechanisms on bacterial cells, such as mechanotransduction, electrotransduction, and magnetotransduction, shedding new light on the mechanisms by which bacteria are activated or inhibited, and therefore allowing to control those mechanisms to obtain desired bacterial response.

## Relevance of Bacterial Cell Microenvironments

Recent studies performed on the effect of physical stimuli in bacterial sensory perception and adaptation has led to the conclusion that bacteria are attuned to mechanical forces, which induces an adaptive behavior of cells toward their surrounding environment (Rusconi et al., 2014). Swimming motility provides a clear example of how bacteria are influenced by the mechanical nature of their surroundings. *Neisseria gonorrhoeae (N. gonorrhoeae*), the bacteria causing gonorrhea, possess protein appendages at their surface, named type IV pili, which enable them to exert physical forces of nanoNewton range on their surroundings, the same amplitude of forces that mammalian cells exert on their own environment (Biais et al., 2010). The forces exerted by *N. gonorrhoeae* cells trigger accumulation of actin and other proteins, events that are critical for the colonization of the host (Howie et al., 2005). More recently, a potential mechanism of action for *E. coli* mechanotransduction has been suggested, indicating that *E. coli* can sense the local mechanical environment through voltage-induced calcium flux, then causing an electric pulse (Bruni et al., 2017).

In fact, it has been proven that bacteria and other mammalian cells such as human sensory neurons, share the electrical pathway as a common tool for sensing their environment. Besides electrical cues, both mammalian and bacterial cells have been reported to sense and respond to different signals, namely magnetic field, electrical field, mechanical, and biochemical cues (Figure 1).

Bacteria are indeed extraordinary organisms, claimed to be one of the dominant forms of life on the planet. It occupies a broad variety of ecological niches on Earth and were the first organisms reported in the fossil record (Rasmussen, 2000). As a single and simple organism, it is remarkable the capacity of bacteria to adapt to different environments, tolerating a big range of temperatures, pressures and pHs, and having the ability to acquire resistance as a mechanism of survival. Their long evolutionary history, being exposed to vastly different physicochemical environments, has made them a multifunctional organism able to detect and respond to a wide range of signals such as chemical, thermal, mechanical, electrical, and magnetic.

This adaptative behavior has been a valuable tool for developing novel strategies for obtaining effective infection control strategies or for potentiating the advantages of beneficial bacterial. By mimicking the bacterial cell microenvironment, the fate of bacterial cells may be tuned for either anti- or pro-microbial approaches.

## The Advent of Antibiotic Resistance

**Figure 2:**
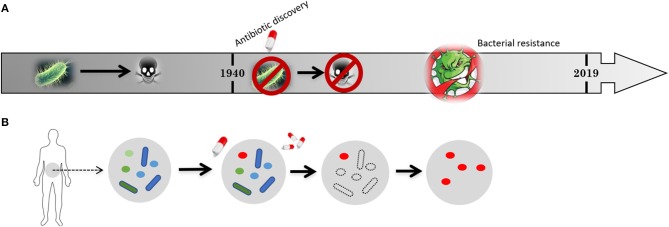
Schematic representation of **(A)** the timeline for the development of bacterial resistance and **(B)** its impact on the natural flora present in human intestine. Previous to penicillin discovery in 1940 bacteria-causing infections killed millions of people but the microbiome of our gut were “untouched” and widely “crowded” by harmless microbes (flora). After 1940, the introduction of antibiotics allowed to cure previously deadly diseases and saved a large amount of lives, extending life span and allowing further medical procedures. Nevertheless, the constant application of antibiotics soon resulted in antibiotic resistant strains. Harmless microbes from the flora in our guts are also killed giving space for the resistant strains to proliferate.

The discovery of penicillin and its introduction in medical practice back in the middle of the last century saved countless lives and had a profound impact on the quality of human life, providing relief from pain and suffering (Miller, 2002). This “wonder drug” eradicated many microbe-causing diseases including tuberculosis and food-borne illness, among many others, indicating the beginning of the antibiotic era (Figure 2A). It seems nowadays difficult to believe that nearly 70 years ago an infected wound could be close to a death sentence.

Nevertheless, persistent use, overuse and misuse of antibiotics worldwide has led to an alarming increased incidence of resistant bacteria (Tenover, 2006; Gould and Bal, 2013; Reardon, 2017). Bacterial infections caused by antibiotic-resistant strains and a lack of new drugs to replace old ones is a growing public health concern, considered by the World Health Organization (WHO) a priority health issue (Collins, 2019). Dangerous bacterial species such as the methicillin-resistant *Staphylococcus aureus* (MRSA) and *vancomycin-resistant enterococci* (VRE) have emerged. If no serious actions are taken in the immediate future, antimicrobial resistance (AMR) may cause many antibiotics to fail, which will become one of the biggest threats to human health (Tenover, 2006; Rasko and Sperandio, 2010; Laxminarayan et al., 2016). Currently, resistant bacteria infect millions of people worldwide and causes more than 750,000 deaths every year, also inflicting enormous costs to health systems. By 2050, it is estimated that more than 10 million people may die per year due to resistant bacteria (Collins, 2019).

It was during Second World War in the 1940s that antibiotics were introduced, saving innumerous wounded soldiers and fast became available for use in the general population. It was then just a matter of time until the resistance to several antibiotics take place. The emergence of clinically antibiotic resistance was early predicted by Alexander Fleming's on his Nobel Prize acceptance speech: “…I would like to sound one note of warning. It is not difficult to make microbes resistant to penicillin in the laboratory by exposing them to concentrations not sufficient to kill them, and the same thing has occasionally happened in the body” (Fleming, 1947). In fact, nowadays, antibiotic resistance is already and should be a public health concern and novel strategies are indeed needed to fight AMR.

Human intestines are home for many different microbes, some of which create resistance to the antibiotics they are exposed to. These resistant strains then spread from person to person, in communities or in hospitals (Figure 2B), ultimately leading to the problem of bacteria resistance. Human body is constituted by 100 trillion cells but only 1 in 10 is actually human. The remaining cells are microorganisms such as bacteria (Relman, 2012). These microorganisms are harmless and live in perfect balance with human body, playing an important role in supporting and maintaining vital functions such as our immune and digestive systems (Relman, 2012). However, when this balance is broken and the delicate ecosystems that bacteria carefully construct in different parts of human body are disrupted, bacteria become pathogenic, causing infection diseases. Pro-microbial approaches thus constitute one strategy that should call the attention of the scientific community. In a broader sense, the equilibrium between anti- and pro-microbial should be an important strategy.

## Anti- and Pro-microbial Equilibrium

Despite being often associated with virulence, infection and disease, bacteria are considered very important microorganisms to sustain human life. They are responsible for the correct functioning of our immune, respiratory, and digestive system (Ichinohe et al., 2011). That is why the right approach to obtain an effective strategy for infection control is to reinforce our beneficial microbial population, the microbiome, in a pro-microbial strategy, while providing an appropriate antimicrobial agent for full eradication of pathogenic bacterial (anti-microbial strategy), without the possibility of developing resistance. The equilibrium between these anti- and pro-microbial approaches is an important twin sustainable strategy for limiting AMR (Jørgensen et al., 2016).

Nevertheless, the dominant strategies to fight AMR have been only focused on drug innovation and the need to “fix the pipeline” of new drugs (Cooper and Shlaes, 2011). Meanwhile, the benefits of microbiome are largely overlooked by the scientific community. The benefits derived from the diversity of beneficial microbes has only recently been proposed (Jørgensen et al., 2016), while antibiotics are more and more considered as non-renewable resources (Cars et al., 2008). Among all antibiotics available on the market nowadays, the most recent class was discovered in the 1980s, which demonstrates the difficulty on the process of finding new effective antimicrobials. It is time to, together with focusing attention on the development of new antibiotics, give more relevance to the diversity of microbes present in the human body that assists on the eradication of harmful bacteria, which means the right balance between anti- and pro-microbial strategies.

In terms of pro-microbial strategies, a technique that is gaining more attention among the scientific community for the treatment and prevention of some infectious disease is the fecal microbiota transplantation (FMT). The process consists in the infusion of beneficial bacteria from the stool of a healthy donor into a recipient with a disease related to an unhealthy gut microbiome (Kim and Gluck, 2019). FMT has been successfully used to treat infections caused by *Clostridium difficile (C. difficile)*, but also tested and recommended for other conditions such as inflammatory bowel disease (IBD), autoimmune disorders, certain allergic diseases, and metabolic disorders such as obesity (Choi and Cho, 2016). The future of infectious disease treatment is thus the promotion of pro-microbial strategies such as FMT. Future challenges regarding this technology are the safety in delivering FMT to patients, being imperative to standardize the methodologies and prepare highly specialized laboratories for stool preparation. Another challenge is to identify the effectiveness of microbiota-based medicines and identify the specific bacteria responsible for this effect.

Regarding anti-microbial strategies, synergistic approaches for inhibiting bacterial pathogenesis, i.e., through the combination of antibiotics and/or other innovative elements that assist the antibacterial effect, drastically reducing the quantity of antibiotic needed for killing bacteria, has been considered as one of the most suitable anti-microbial strategies for effectively kill harmful bacteria (Fayaz et al., 2010). Such strategies cause less evolutionary stress on bacteria population and thus prevent the emergence of resistance mechanisms. The increasing understanding of bacterial pathogenesis and intercellular communication in a broader sense, both from the physical and biochemical points-of-view has been a valuable tool to develop new strategies that meets these challenges (Hajipour et al., 2012).

**Figure 3:**
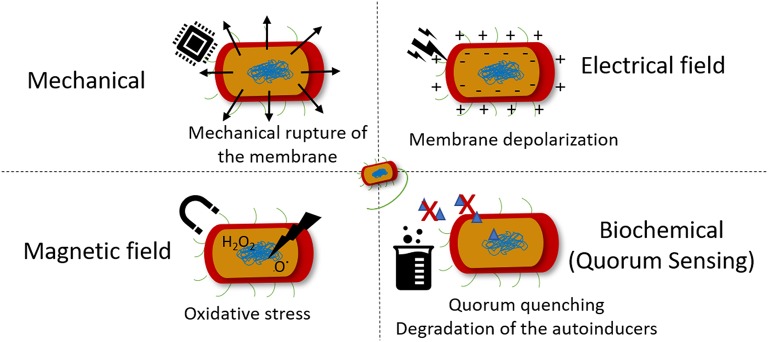
Stimuli that bacteria sense and the mechanism of action of each bactericidal effect.

Taking into consideration that bacteria can indeed feel the surrounding environment and modify their phenotype in response to it, the main cues responsible for the tailoring of bacteria behavior and thus assist the action of antibiotics are depicted in Figure 3. Several studies have been performed on the effect of specific stimuli on bacteria, namely the mechanical, magnetic, electrical, and biochemical [quorum sensing (QS) mechanism] cues. Their effects on bacterial cell, advantages and disadvantages are summarized in Table 1.

**Table 1:** Summary of the effects of different physical and biochemical stimuli on bacteria.

**Stimulus**	**Effect on bacterial cell**	**Advantages**	**Disadvantages**	**Common features**
Mechanical	Membrane disruption (Hazan et al., 2006; Chen et al., 2010)	Antifouling/Antibiofilm (Mah and O'Toole, 2001; Hazan et al., 2006; Paces et al., 2014)	In the case of ultrasound, the acoustic cavitation of microbubbles in the blood may cause rupture of blood vessels (Chen et al., 2010)	
Magnetic	Interfere with ion transport in the membrane/membrane rupture (Worcester, 1978)	Possibility of remote stimulus (Dobson, 2008; Guduru and Khizroev, 2014) Oxidative stress (ROS formation)-based killing effect (Ghodbane et al., 2013)	Surrounding temperature may increase—promoting eukaryotic cell death (Ghodbane et al., 2013)	Possibility of using in synergy with commonly used antimicrobials (Qian et al., 1997; Rediske et al., 1999; Justin and Thomas, 2012)Decreased virulenceReduced risk of drug resistance
Electric	Electro permeabilization or irreversible electroporation (Valič et al., 2003, 2004)	Oxidative stress (ROS, H_2_O_2_ and RNS formation)-based (Valič et al., 2004; Boda and Basu, 2017) Possibility of triggering proliferation of bacterial cells (Ueshima et al., 2002; Carvalho et al., 2019)	Requires the application of an electrical field on bacterial solution—not recommendable for *in vivo* applications (Boda and Basu, 2017)	
Bio-chemical	Chemotaxis (Mao et al., 2003)	Effectiveness of antibiotics for killing pathogenic bacteria	Possibility of developing resistance	
	Quorum quenching (Hentzer and Givskov, 2003; Roche et al., 2004; Ni et al., 2009; Rutherford and Bassler, 2012)	QS autoinducers degradation (Ivanova et al., 2013, 2015a,b,c) Attenuates virulence (Ivanova et al., 2013, 2015c)	Endogenous stimuli (the stimuli needs to be applied internally)	

### Mechanical Cues

The effect of mechanical vibrations on bacteria surface adhesion, proliferation and virulence has been mainly evaluated for the inhibition of biofilm formation, a protective mode of growth that confer pathogenic bacteria increased resistance to conventional antibiotics and host defenses mechanisms (Mah and O'Toole, 2001). Surface acoustic waves generated from electrically activated piezo elements has been reported to be repulsive to bacteria and interfere with the docking and attachment of planktonic microorganisms to solid surfaces (Hazan et al., 2006). Also, vibration loads generated by magnetoelastic materials, which possess magnetostrictive properties, converting a magnetic stimulus into a mechanical deformation, were found to significantly reduce the adherent bacteria on samples exposed to different types of microorganisms (Paces et al., 2014).

The use of ultrasounds have also been used as a mean for preventing biofilm formation. The application of ultrasound induce an acoustic cavitation phenomenon, which results in the formation of cavitation bubbles that implode and generate shock waves that cause mechanical damage to the bacteria and the formation of free radicals that creates oxidative stress to bacteria (Figure 3) (Gera and Doores, 2011). To obtain a relevant reduction on bacterial counts, ultrasound has been applied in combination with antibiotics as a synergistic approach. *Pseudomonas aeruginosa (P. aeruginosa)* and *E. coli* biofilms were thus efficiently eradicated by gentamicin sulfate in combination with ultrasound (Qian et al., 1997; Rediske et al., 1999). Also, the killing effect of ZnO NPs against *Staphylococcus aureus* (*S. aureus*) and *P. aeruginosa* was boosted when ultrasound was applied to the medium (Justin and Thomas, 2012). It is worth mentioning that the effect of the mechanical damage created by the acoustic cavitation of microbubbles in the blood, the possibility to induce rupture of blood vessel walls and interfere with blood flow may limit the application of this type of strategies *in vivo* (Chen et al., 2010).

### Magnetic Cues

The influence of magnetic field on biological systems, namely on biomolecules, cells and living organisms, is an important field of research due to the emergence of electromagnetic pollution in the form of low and high radio frequency magnetic fields from electronic devices (Boda and Basu, 2017). It is believed that magnetic field interfere with the mechanism of ion transport via membrane channel proteins, leading to osmotic imbalance and membrane rupture. These phenomenon is attributed to the diamagnetic anisotropy exhibited by the large number of membrane proteins on bacteria cell, which result from the axial alignment of peptide bonds and specific amino acid residues containing aromatic groups (Worcester, 1978). Therefore, the lipids and ion channel proteins that are present in bacterial membrane undergo conformational changes that lead to the dysfunction of these proteins, disrupting essential ion transport mechanisms on bacteria. One of the first reports of this effect on bacteria proved that magnetic field cause rotational motion of ion-protein complexes leading to the escape in *E. coli* (Binhi et al., 2001). Both low frequency electromagnetic fields and moderate intensity static magnetic fields have been proven to induce bacteriostatic and/or bactericidal effects (Li and Chow, 2001). The generation of free radicals upon application of a magnetic field, leading to bacterial cell oxidative stress and genotoxicity (Figure 3) has been another proposed mechanism of action involved in bacterial killing and possibly mammalian cell damage (Ghodbane et al., 2013). In fact, the potential mutagenicity and carcinogenic effect of low and high frequency magnetic fields on mammalian cells/tissues has been poorly investigated (Ikehata et al., 1999) and the studies often result on contradictory information. Further studies need to be performed to assess the potential harm these approaches might induce to biological systems.

### Electrical Cues

It is known that direct application of strong electric fields may be bactericidal or a mean of preventing device-related infections, which are caused by biofilm formation, or even to disinfect contaminated liquids (Poortinga et al., 2001; van der Borden et al., 2005; Hong et al., 2008; Istanbullu et al., 2012; Gall et al., 2013). Moreover, the ability of electric fields to promote wound healing through angiogenesis while reducing microbial bioburden at the surface of material has already been proven (Asadi and Torkaman, 2014).

The electrical-based killing mechanism of action involves an increase in cell membrane permeability, known as electropermeabilization or irreversible electroporation. This occurs when the induced transmembrane voltage exceeds the threshold transmembrane membrane voltage (200–1,000 mV), while the resting transmembrane potential ranges between −20 and −200 mV for most cells (Valič et al., 2004). For keeping the resting membrane potential nearly constant, the Na/K pump actively exudes three Na^+^ for every two K^+^ pumped into the cell. The perturbation of these ion concentrations can lead to hyperpolarization, wherein the membrane potential becomes more negative or depolarization and the membrane potential becomes less negative toward zero (Valič et al., 2003). The electropermeabilization effects from the application of low-strength electric fields has been shown to boost the effect of antibiotics against bacteria in biofilms, diminishing the concentration of antibiotic needed to kill bacteria (Costerton et al., 1994). On the other hand, high strength electric pulses resulted in an efficient bactericidal effect on both gram-positive and gram-negative bacteria. These strategies however require the direct application of an electrical field on bacterial solution, which may not be recommendable for *in vivo* applications. Similarly to other stimuli previously mentioned the formation of reactive oxidative species (ROS) such as hydrogen peroxide (H_2_O_2_) and reactive nitrogen species (RNS) was also indicated as a possible mechanism of action for the bactericidal effect of low strength electric field (Boda and Basu, 2017).

Electrical stimulus has been mainly reported to impart bactericidal and bacteriostatic effect rather than a proliferation effect. Nevertheless, it has been also reported that low frequency mechanical stimuli leading to surface charge variations, induce similar effect to those occurring with eukaryotic cells, i.e., proliferation, being the effect dependent on the applied physical stimuli conditions (Ueshima et al., 2002).

### Biochemical Cues

**Figure 4:**
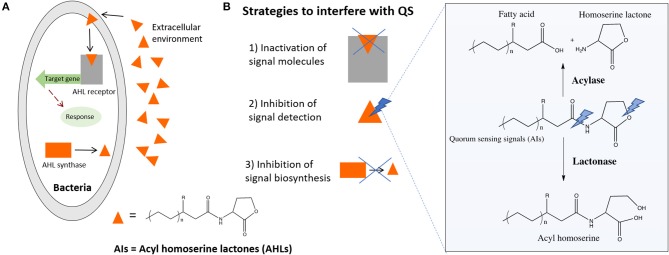
**(A)** Simplified QS system of Gram-negative bacteria, general chemical formula of the signaling molecules and **(B)** strategies to QQ including the enzymatic degradation of AHL signals by AHL-lactonase and AHL-acylase.

Besides chemotaxis, another important example of how bacteria sense its environment is the QS mechanism they use to express virulence factors, allowing bacteria to regulate community-wide behaviors including biofilm formation, virulence, conjugation, sporulation, and swarming motility (Rutherford and Bassler, 2012). This mechanism of cell-to-cell communication is based on the production, secretion, and detection of small signaling molecules, called autoinducers (AIs). In the QS-regulated communication, bacteria secrete signaling molecules, the AIs that are further recognized by specific receptors, allowing bacteria to act collectively as a multicellular microorganism. This knowledge is being increasingly used to develop new strategies for infection control (Figure 4A). The inactivation of the QS signals in a process called quorum quenching (QQ) is an innovative strategy to control bacterial infections (Hentzer and Givskov, 2003; Roche et al., 2004; Ni et al., 2009; Rutherford and Bassler, 2012). Brominated furanones interfere with QS by acting as antagonists to receptors (Rasmussen and Givskov, 2006; Kociolek, 2009). Similarly, enzymes such as acylase and lactonase have been shown to selectively degrade N-Acyl homoserine lactone (AHL) signals of Gram-negative bacteria (Dong et al., 2000, 2001) (Figure 4). In line with this knowledge, acylase has been successfully used to coat indwelling medical devices through functionalization techniques such as layer-by-layer technique. The enzyme multilayer coatings significantly reduced the bacterial load and biofilm formation on functionalized silicone-based urinary catheters, assessed with an *in vitro* catheterized bladder model (Ivanova et al., 2015b) and *in vivo* using an animal model (rabbit), where the QQ and matrix degrading enzyme assemblies delayed the biofilm growth up to 7 days (Ivanova et al., 2015a). Moreover, the QQ enzyme coatings were fully biocompatible since they were tested with mammalian cells such as fibroblasts over 7 days, the extended useful life of urinary catheters, and no toxicity was observed.

The main advantage of this approach is that it attenuates virulence, exerting less selective pressure on bacteria and reducing the risk of resistance development to drugs. Moreover, it affects bacterial behavior but does not kill or inhibits their growth, thus allowing the host defense system to eliminate attenuated bacteria or substantially increase the effect of co-administered antibiotics (Ivanova et al., 2013, 2015c). The action of such enzymes (Figure 4B) creates the conditions to eradicate the infection by the natural host immune system before virulence is established.

## Smart Materials and Active Surfaces for Tailoring Bacterial Responses

The application of a magnetically and electrically active microenvironment is a strategy that has been widely explored in mammalian cells and that can be also used for tailoring specific bacterial responses. It is well-stablished that electroactive materials such as piezoelectric polymers and magnetoelectric composites develop voltage variations at the surface of the material when a mechanical stress (Ribeiro et al., 2015) or a magnetic field (Ribeiro et al., 2016), respectively, is applied, thus promoting the adhesion and proliferation of eukaryotic cells, such as osteoblasts. Knowing that bacteria are also able to sense these types of stimuli, these materials seem to constitute a suitable approach for both anti- and pro-microbial applications by developing active surfaces based on those materials. Examples of such materials are the ones possessing mechanoelectric, magnetostrictive, and magnetoelectric properties.

**Figure 5:**
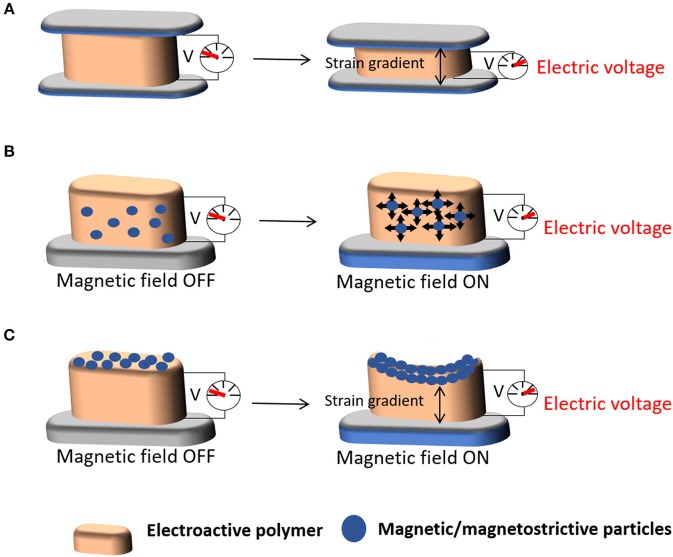
Schematic representation of the **(A)** mechanoelectric properties of a material upon the application of mechanical stimuli and **(B,C)** magnetoelectric properties of scaffolds upon the application of magnetic stimuli.

Mechanoelectric materials are materials mainly constituted by, for example, piezoelectric polymers that respond to a mechanical stimulus, inducing an electrical charge variation in the material (Figure 5A). Magnetic and magnetoelectric materials are composites comprising magnetic or magnetostrictive particles and a piezoelectric polymer. Due to their magnetic component, they sense a magnetic field that induce a mechanical stimulation on the material, due the incorporated magnetic or magnetostrictive properties, which further induce an electrical polarization variation due to the piezoelectric phase present in the composite (Figures 5B,C). These materials thus respond to different stimuli, namely mechanical and magnetic field. Both stimuli are induced with the help of a bioreactor that provides specific cues on the materials and thus on the cells, for cell response investigation studies, or on active coatings through the surface functionalization of materials where those stimuli are present or can be induced, as for example in biomedical devices.

Piezoelectric synthetic polymers are the most widely used in the development of mechanoelectric materials for biomedical applications. Poly(vinylidene fluoride) (PVDF) and vinylidene fluoride (VDF) copolymers possess high electroactive properties, including piezoelectric, pyroelectric, and ferroelectric properties, which makes them particularly used (Dubois, 1996; Serrado Nunes et al., 2009). Despite the fact that PVDF and its copolymers are not biodegradable, they are biostable and thus widely used. Nevertheless, for biomedical applications, polymers must satisfy several requirements, including biocompatibility, biostability, and/or biodegradability to non-toxic products (Rezwan et al., 2006). That is why poly(L-lactid acid) (PLLA) (Preethi Soundarya et al., 2018), poly(3-hydroxybutyrate-co-3-hydroxyvalerate) (PHBV) (Chen and Wu, 2005; Amaro et al., 2018), and collagen, a piezoelectric natural polymer (Zhou et al., 2016), are being increasingly used as electroactive polymers since they combine piezoelectricity with biodegradability.

On the other hand, magnetic nanocomposites with magnetoelectric properties may be obtained by adding nanomaterials such as pure metals (Co, Fe, Ni) and metal oxides (iron oxides Fe_2_O_3_ or Fe_3_O_4_ and ferrites such as BaFe_12_O_19_ and CoFe_2_O_4_) combined with the above-mentioned piezoelectric polymers (Kudr et al., 2017; Fernandes et al., 2018). The obtained magnetic nanocomposites are very interesting materials since they allow to remotely mechanical and/electrically stimulate tissues from outside of the human body (Dobson, 2008; Guduru and Khizroev, 2014) and for specific cell cultures in bioreactors (Ribeiro et al., 2016). The possibility to remotely control tissue stimulation without the need of patient movement is certainly an innovative approach and is regarded as a breakthrough platform for tissue engineering applications (Silva et al., 2013; Ribeiro et al., 2016). In fact, the magnetic actuation ability of the magnetoelectric composite allows the mechanical and electrical stimuli of neighboring cells (Martins and Lanceros-Méndez, 2013).

**Figure 6:**
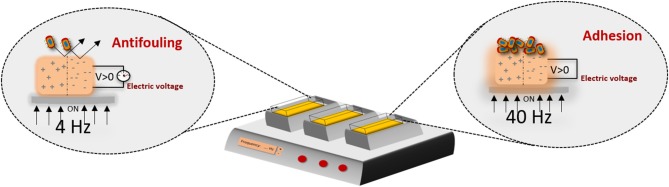
Electroactive microenvironments created by a mechanical bioreactor on a piezoelectric scaffold, inducing different responses on bacterial cells including proliferation or growth inhibition/antifouling properties, depending on the frequency applied, thus proving a new concept of bacteria susceptibility to physical stimuli. Such approaches are important to further define suitable anti- and pro-microbial strategies, intended for pathogenic and functional bacteria, respectively (Carvalho et al., 2019).

In microbiology this approach could also be valuable, for example, for the prevention of infection of orthopedic indwelling devices by external stimulation. To obtain such effect, one can use the potential of electrically and magnetically active materials/scaffolds. As previously mentioned, the development of these kind of materials has been explored in tissue engineering but poorly investigated in microbiology. The effect of a strong electrical field on the bacteria behavior, rather than acoustic mechanic waves, has been reported in a study where the effect of a piezoelectric material (ceramics) on bacteria was performed and the killing effect was due to the formation of ROS (Tan et al., 2016). On the other hand, the proliferation effect of bacteria has been observed at the surface of electrically polarized hydroxyapatite (Ueshima et al., 2002). Recent findings reported that bacterial cells behavior, which grow upon piezoelectric polymers, may be tailored according to the surface charge of the material and on the application of weak electrical field, promoted by a piezoelectric polymer under mechanical stimuli, demonstrating a different behavior between Gram-positive and Gram-negative cells (Carvalho et al., 2019). The Gram-positive bacteria seems not adhere to positively charged surface, as opposed to the negatively charged surface. In the presence of an electrical stimuli, this strain shows a different behavior: the lower frequency promotes the antifouling and the higher stimulates the bacteria adhesion (Figure 6) (Carvalho et al., 2019).

## Concluding Remarks and Future Perspectives

The ambition to create novel strategies to fight bacteria resistance using physical stimuli is an attractive and valid approach that relies on the fact that bacteria sense their environment and respond to it. This review thus calls the attention for the use of innovative electroactive smart materials as a novel tool for tailoring bacteria behavior and thus fight bacteria resistance using, not only anti-microbial strategies, but also pro-microbial ones. The most attractive feature of using such materials is the possibility for triggering the inhibition and proliferation of bacteria by changing the conditions applied, when bioreactors or smart and responsive surfaces and coatings are used. From one side, defining the conditions for antimicrobial strategies will allow these materials to be used synergistically with commonly applied antibiotics or other innovative elements that assist the antibacterial effect, reducing the quantity of antibiotic needed for killing bacteria. Such strategies cause less evolutionary selective pressure on bacteria population and thus prevent the emergence of resistance mechanisms. On the other side, defining the conditions for proper bacteria proliferation approaches will be the opportunity to pursue a pro-microbial activity, potentiating the function of human microbiome in assisting vital functions in our body. A healthy microbiome is essential for human and animal well-being since it has the ability to educate the immune system and keep the pathogenic bacteria out, apart from the obvious role it has on the digestive system.

Therefore, novel materials or coatings that assist the antimicrobial effect and thus prevent the occurrence of nosocomial infections in clinical settings (due to the contamination of medical devices such as stents and catheters or medical textiles) are very appealing and needed to be used as first line defense against pathogenic bacteria, while potentiating the action of beneficial bacteria will allow to reinforce the microbiome, essential for disease prevention. More importantly, the application of electroactive materials may thus be the future for developing smart implantable devices, which due to their electric-sensitive properties may be used not only to promote anti- and pro-microbial strategies but also to take advantage of their characteristics for sensor applications.

## Author Contributions

All authors listed have made a substantial, direct and intellectual contribution to the work, and approved it for publication.

### Conflict of Interest

The authors declare that the research was conducted in the absence of any commercial or financial relationships that could be construed as a potential conflict of interest.
